# Co-Designing Priority Components of an mHealth Intervention to Enhance Follow-Up Care in Young Adult Survivors of Childhood Cancer and Health Care Providers: Qualitative Descriptive Study

**DOI:** 10.2196/57834

**Published:** 2025-04-25

**Authors:** Sharon H J Hou, Brianna Henry, Rachelle Drummond, Caitlin Forbes, Kyle Mendonça, Holly Wright, Iqra Rahamatullah, Perri R Tutelman, Hailey Zwicker, Mehak Stokoe, Jenny Duong, Emily K Drake, Craig Erker, Michael S Taccone, Liam Sutherland, Paul Nathan, Maria Spavor, Karen Goddard, Kathleen Reynolds, Fiona S M Schulte

**Affiliations:** 1 Faculty of Education Simon Fraser University Burnaby, BC Canada; 2 BC Children's Hospital Vancouver, BC Canada; 3 Department of Oncology University of Calgary Calgary, AB Canada; 4 Department of Psychology University of Calgary Calgary, AB Canada; 5 Faculty of Health Dalhousie University Halifax, NS Canada; 6 Department of Pediatrics Dalhousie University Halifax, NS Canada; 7 Childhood Cancer Survivor Canada Toronto, ON Canada; 8 Community Health Sciences Cumming School of Medicine University of Calgary Calgary, AB Canada; 9 Department of Pediatrics University of Toronto Toronto, ON Canada; 10 Institute for Health Policy, Management and Evaluation University of Toronto Toronto, ON Canada; 11 Department of Pediatrics Faculty of Medicine and Dentistry University of Alberta Edmonton, AB Canada; 12 Department of Surgery Faculty of Medicine The University of British Columbia Vancouver, BC Canada; 13 Department of Family Medicine, Cumming School of Medicine University of Calgary Calgary, AB Canada; 14 Long Term Survivors Clinic, Alberta Children's Hospital Calgary, AB Canada

**Keywords:** mobile health, mHealth, pediatric oncology, cancer survivorship, qualitative research, patient-oriented research, co-design, intervention development

## Abstract

**Background:**

Survivors of childhood cancer are at risk of medical, psychological, and social late effects. To screen for their risks, receipt of consistent, cancer-specific follow-up care is crucial. However, <50% of survivors attend their aftercare, and only 35% of them recognize that they could have a serious health problem. The use of mobile health (mHealth) is a promising form of intervention to educate, connect, and empower survivors of childhood cancer on the importance of follow-up care.

**Objective:**

This study aimed to use co-design to identify the priority components to include in an mHealth intervention with young adult (aged between 18 and 39 years) survivors of childhood cancer and health care providers.

**Methods:**

This study was conducted between January and November 2022 in Canada and used patient-oriented research methods. Participants were recruited through local or provincial long-term follow-up clinics, using convenience sampling from patient partners who assisted in recruiting survivors across geographical areas in western, central, and eastern Canada, and social media outreach (X, formally known as Twitter; Facebook; and Instagram). Qualitative descriptive data (focus group interviews) from survivors of childhood cancer and health care providers (individual interviews) were gathered. We analyzed the collected data using reflexive thematic analysis and verified it through member checking techniques through an online community engagement event.

**Results:**

We conducted with patient partners 5 online (Zoom) focus groups with 22 survivors of childhood cancer (mean age 29.19, SD 4.78 y). We conducted individual telephone interviews with 7 health care providers. Participants identified five priority areas to be included in an mHealth intervention: (1) connections, (2) education and information, (3) engagement, (4) personalization, and (5) resources. Results were shared with and validated by survivors of childhood cancer, their families, health care providers, and academic researchers as part of a community engagement event. Small and large group discussions were facilitated to allow participants to review and discuss the accuracy of the themes derived regarding the core components to be included in mHealth. A graphic recording artist visually captured key ideas from the event. A subset of the participants also completed a web-based satisfaction survey, and responses indicated that the community engagement event was generally well received.

**Conclusions:**

Results from this study have provided the necessary foundation to progress in intervention development. The next step of this multiphased project is to build an innovative and accessible mHealth intervention prototype that is based on the identified core components and is grounded in an established conceptual framework for co-design of mHealth.

## Introduction

### Follow-Up Care for Survivors of Childhood Cancer

Due to advances in treatment and supportive care, the survival rate for pediatric cancer (age 0-19 years) now exceeds 80% [1]. In turn, the number of long-term survivors of childhood cancer has grown exponentially, with more than 45,000 survivors living in Canada today [2]. While this is encouraging, survivors of childhood cancer are at a lifelong risk of developing medical, psychological, and social late effects due to their cancer treatment [3]. By the age of 50 years, nearly 100% of survivors develop one or more chronic health conditions, many of which are disabling or life-threatening [4,5]. This prevalence suggests an important need for early screening and intervention of late effects to improve longer-term outcomes for this group considered vulnerable.

Consistent, cancer-specific follow-up care is associated with better long-term outcomes, including earlier identification of late effects or secondary cancers and minimized risk of morbidity and mortality [6-8]. However, less than 50% of survivors of childhood cancer attend long-term follow-up clinics [9,10]. Known barriers to attending follow-up care in Canada include limited knowledge of late effects and recommendations for follow-up care, treatment factors (eg, diagnosis and type of treatment), distance from cancer-specific follow-up clinics, and sociodemographic factors (eg, minority status and male sex) [7,10]. Furthermore, only 35% of survivors recognize that they could develop a serious health problem [11]. Young adults (aged between 18 and 39 years [12]) survivors of childhood cancer are especially at risk of not receiving consistent long-term follow-up care due to their unique developmental stage. Specifically, this is a critical and dynamic time during which major transitions occur, including greater autonomy, independent living, and financial independence, as well as a period of increased mental health concerns and risk-taking behaviors [13]. The transition from pediatric to adult health services is also particularly challenging and may result in diminished engagement in follow-up care [14].

### Mobile Health Intervention for Survivors of Childhood Cancer

Development of innovative interventions is needed to better educate and engage survivors of childhood cancer in their follow-up care. An ideal intervention would meet survivors where they are, be accessible outside of formal follow-up care programs, account for the sociocultural context of survivors [15], and ultimately address the distinct needs and challenges of this population. Mobile health (mHealth) refers to the use of wireless technology in medical care to deliver health education. mHealth has shown potential to educate patients about preventative health care [16]. Furthermore, mHealth may serve to break down some of the geographic barriers and issues related to accessibility faced by survivors of childhood cancer residing in more remote or rural regions [17]. Given the widespread use of smartphones among young people [18], mHealth has the potential to address the unmet psychosocial and health care needs of young adult survivors of childhood cancer. Therefore, the use of mHealth is a promising new form of intervention to educate, connect, and empower survivors of childhood cancer on the importance of cancer-specific follow-up care.

mHealth interventions targeting young adult survivors of childhood cancer are in their infancy [19,20]. A systematic review of eHealth and mHealth interventions in pediatric cancer lends support for the feasibility and acceptability of technology-based approaches to improve outcomes of children with cancer. However, evidence of the effectiveness of interventions targeting specific outcomes (eg, emotional distress and health behaviors) remains mixed [19]. Limitations of existing interventions include restrictions to single-site pretest-posttest designs, a failure to consider an iterative process to intervention development, and a lack of engagement with patients as partners in the co-design of these programs, and intervention components must be guided by survivors’ priorities [21]. Engaging patients as partners is a feasible and efficient way to conduct clinical research, as they are considered experts through their own lived experiences [21]. Research shows that patient engagement in health research has many benefits, including higher participation rates, design of study protocols with more relevant outcomes, and more meaningful and accessible means of disseminating research to study participants and community members [21]. However, survivors are seldom included in the development of interventions directed at improving knowledge of the importance of long-term follow-up care. Therefore, co-designing with survivors of childhood cancer in addressing their own barriers to attending follow-up care may be a promising means to enable survivors of childhood cancer to understand and engage in their follow-up care and maximize their outcomes. There is also limited research that incorporates health care provider perspectives [22,23]. Health care providers that deliver follow-up care to survivors of childhood cancer may offer complementary insights on the mHealth intervention development in the context of Canadian health care systems.

### Current Research

The overarching goal of this research was to co-design an mHealth intervention with young adult survivors of childhood cancer, as well as health care providers that deliver follow-up care to this population. In this research, we refer to co-design as a meaningful engagement with end users in the entirety of the research process [24]. In addition, our research approach adhered to the best practices outlined by the Strategy for Patient-Oriented Research (SPOR) of the Canadian Institutes of Health Research (CIHR) [25].

As a foundational step toward mHealth intervention development, we conducted a qualitative study to understand and identify the priority components to be included in an mHealth intervention that can engage, educate, and empower survivors of childhood cancer on the importance of attending their follow-up care. Subsequently, we validated the results by seeking feedback from multiple informants, including survivors, caregivers, health care providers, and researchers, through a community engagement event.

## Methods

### Overview

This study was conducted as part of a larger, multiphased project regarding survivors of childhood cancer and their follow-up care experiences [26,27]. We used patient-oriented research and qualitative descriptive study design. Qualitative description is a qualitative research framework that provides detailed experiences directly from participant perspectives [28]. This study design is well suited for health sciences research because it captures broad insights and helps address key clinical and health care services questions [29].

Study data were collected and managed using REDCap (Research Electronic Data Capture; Vanderbilt University) electronic data capture tools hosted at the University of Calgary. REDCap is a secure, web-based software platform designed to support data capture for research studies, providing (1) an intuitive interface for validated data capture, (2) audit trails for tracking data manipulation and export procedures, (3) automated export procedures for seamless data downloads to common statistical packages, and (4) procedures for data integration and interoperability with external sources [29,30].

### Ethical Considerations

Ethics approval for this study was granted by the Health Research Ethics Board of Alberta—Cancer Committee (HREBA.CC-20-0248). As this project involved human participants, informed consent was obtained from all participants. Data collected were deidentified before data analysis. Participants were compensated for their time in the study (refer to the Procedure section for compensation details).

### Patient and Public Involvement

In line with best practices of CIHR SPOR [25], we have reported the background, objectives, methods, and results of this project based on the checklist from the Guidance for Reporting Involvement of Patient and the Public Short Form [31]. Patient partners were involved as coresearchers throughout the project, including study design, recruitment, data collection, interpretation of results, and knowledge dissemination. Patient partners were compensated financially for their time.

### Participants

Survivors were eligible to participate in the qualitative study if they were (1) currently aged between 18 and 39 years, (2) diagnosed with cancer before the age of 18 years, (3) at least 5 years after diagnosis and/or 2 years after treatment, and (4) currently living in Canada. Health care providers were eligible to participate in the study if they were (1) delivering clinical care to survivors of childhood cancer, (2) practicing for >5 years, and (3) currently living in Canada.

Participants were eligible to participate in the community engagement event if they were (1) survivors of childhood cancer as defined earlier, (2) parents or caregivers of survivors of childhood cancer, (3) health care providers that provide follow-up care to survivors of childhood cancer as defined earlier, or (4) researchers of childhood cancer survivorship.

### Recruitment

Several strategies were used to recruit individuals from diverse sociodemographic backgrounds as part of the larger, multiphased project [26]. First, survivors and health care providers were informed about the study by their local or provincial long-term follow-up clinics, where they were given the option to complete a consent-to-contact form and subsequently invited to participate by the study team. Second, patient partners assisted in recruiting survivors across geographical areas in western, central, and eastern Canada. The practice of patients recruiting patients to integrate diverse patient perspectives is supported by the principle of inclusiveness according to the CIHR SPOR framework [25]. Third, social media outreach was used, including X (formerly known as Twitter; X Corp), Facebook (Meta Platforms), and Instagram (Meta Platforms). This strategy involved recruiting through patient and young adult cancer advocacy groups and communities by sharing study graphics (eg, Childhood Cancer Survivor Canada and #AYACSM).

Participants that completed other phases of this project were invited to participate in this qualitative study. Participants that participated in other phases of the project, as well as those who did not participated in the project, were all welcome to attend the community engagement event.

### Procedure

We conducted 5 online focus groups with survivors of childhood cancer on Zoom (Zoom Communications Inc) between March and July 2022. Focus group discussions lasted approximately 90 minutes. Eligible participants were contacted and provided with a link to consent to the study on the web through REDCap. They were then provided with a Zoom link and date for the focus group. Participants were compensated with a CAD $25 (US $17.91) e-gift card for their participation. Similarly, we conducted telephone interviews with health care providers who followed the same consent process as survivor participants. Each interview lasted 15 to 20 minutes. Health care providers were also compensated with a CAD $25 (US $17.91) e-gift card for their participation.

We hosted a half-day online community engagement event via Zoom in October 2022. The event was cofacilitated by 2 academic researchers (FSMS and SHJH) and 2 patient partners (RD and IR). A collaborator from a digital technology company, Cambian, attended the event to provide input from a feasibility and usability standpoint. Cambian specializes in providing collaborative health care information services. A graphic recording artist from Fuselight Creative attended the event to visually capture central and representative ideas generated from the community event. Fuselight Creative is a visual facilitation company that specializes in the strategic use of graphic recording to enhance interactive engagement and learning in community events.

Eligible participants were asked to register for the event and provide their email address to receive the Zoom link. Registered participants provided verbal consent to partake on the day of the event on the web via Zoom. Participants were compensated with a CAD $25 (US $17.91) honorarium for their participation in the community engagement event. Likewise, patient partners (RD and IR) were compensated for their cofacilitation of the event.

### Measures

#### Sociocultural Demographics

For the qualitative study, survivors completed an initial questionnaire regarding their sociodemographic background, including age, sex, gender, relationship status, family composition, and race and ethnicity. Survivors also completed questions regarding their clinical history, such as their diagnosis, age at diagnosis, type of treatments received, and number of years after treatment. Health care providers completed a similar questionnaire to survivors regarding their sociodemographic background, as well as questions regarding their professional experiences, including the number of years practicing and years of experience delivering care to survivors of childhood cancer.

#### Interview Guide

Survivors engaged in an online focus group discussion, guided by a semistructured interview guide exploring the development of an mHealth intervention for survivors of childhood cancer to optimize their engagement in follow-up care. Questions were codeveloped with patient partners and centered on the development of content (eg, “What are your thoughts on creating a platform for survivors?”), education (eg, “What type of information would you want included?”), communication (eg, “Would you want an opportunity to interact with other survivors and/or healthcare providers?”), and engagement (eg, “What are some features that could help you engage in your follow-up care?”) related to the mHealth intervention.

Health care providers engaged in an individual telephone interview. The same set of semistructured interview questions was administered, with wording modified to reflect the health care providers’ perspective on the same topic. The complete set of questions asked during the focus groups can be found in Multimedia Appendix 1.

#### Small and Large Group Discussions

As part of the community event, participants engaged in interactive small and large interactive group discussions. Facilitators posed semistructured questions on the accuracy, representativeness, and resonance of the themes conceptualized from the data. During small group discussions (3-4 participants per group), differences in perspectives were discussed in the presence of an observer who was a member of the research team. The observer’s role is to ensure equal participation among members and that no one individual or group’s views are dominant over others. The observer takes notes, facilitates the flow of conversation and turn-taking among participants, and shares a summary of key discussion points with members to ensure the accuracy of notes taken. Key discussion points from the small group sessions were subsequently integrated into the larger group discussion (all participants) to achieve consensus, facilitated by 2 main facilitators (SHJH and FSMS) and 2 patient partners (RD and IR). Large group discussions were facilitated using interactive media features, including a Google Jamboard, Mentimeter, and polling on Zoom.

#### Satisfaction Survey

Participants completed a web-based questionnaire within 1 week following the event. Questions were derived and adapted from the Public and Patient Engagement Evaluation Tool [32]. Participants were asked to rate the extent to which they agreed with statements regarding their experience at the event, using a 5-point Likert scale (1=strongly disagree and 5=strongly agree). Questions were centered on participants’ understanding of the nature of the workshop (eg, “I had a clear understanding of the purpose of this community workshop”), as well as whether the workshop met the stated objectives described herein. We also solicited feedback from participants using an open-text response format.

#### Analysis Plan

Sociodemographic information gathered from participants was summarized using descriptive statistics and analyzed with SPSS (version 28.0; IBM Corp). Qualitative descriptive data gathered from focus groups and interviews were audio-recorded, transcribed verbatim, and deidentified. Transcripts were subsequently analyzed using reflexive thematic analysis [33]. Reflexive thematic analysis is a theoretically flexible analytical approach used to identify patterns of meaning and conceptualize them into themes. This type of analysis is commonly used in qualitative description studies [34]. In total, 5 researchers (SHJH, BH, RD, KM, and HW) conducted the analysis according to the steps outlined by Braun and Clarke [33]. First, researchers read the transcripts several times to familiarize themselves with the data. Next, researchers developed a preliminary coding framework and coding book. Each researcher independently coded the same transcript and met as a team to compare coding choices and ensure alignment in the interpretation of the codes. Following this, the remaining transcripts were each analyzed by 2 researchers. Theme abstraction took place iteratively among the researchers by meeting regularly to discuss and review each researcher’s interpretations. Any differences in theme abstraction were reconciled through open dialogue until a consensus was reached. Data analysis was supported using NVivo (version 14; Lumivero) [35], a qualitative data management software. Multimedia Appendix 2 includes a reflexivity statement from all researchers involved in the thematic analysis.

Member checking, also known as response validation, is a validation technique used in qualitative health research and has been recognized as a method of rigor to enhance the trustworthiness of qualitative data [36]. Member checking was conducted with multiple informants, including survivors, caregivers, health care providers, and researchers, through an online community engagement event. Member checking was achieved through multiple methods, including small and large group discussions, satisfaction surveys, and graphic recording throughout the community engagement event.

## Results

### Participant Characteristics

In total, we conducted 5 online focus groups with 22 survivors of childhood cancer, with a range of 4 to 6 participants present per focus group. The mean age of survivors was 29.19 (SD 4.78) years. Over 95% (21/22) of survivors indicated that their assigned sex was female and likewise that their gender identity was female. Most survivors (19/22, 86%) identified as White. Survivors came from 6 provinces, most prevalently from Alberta (10/22, 46%), Ontario (5/22, 23%), and Nova Scotia (4/22, 18%). Most survivors (18/22, 82%) lived in an urban geographical region. Survivors reported a history of leukemias (11/22, 50%), lymphomas (6/22, 27%), and solid tumors (5/22, 23%) as the most common diagnoses. The average age at diagnosis was 10.59 (SD 5.45) years, and the mean time off treatment was 17.45 (SD 6.81) years.

We conducted individual telephone interviews with 7 health care providers. In total, 6 health care providers indicated that their sex assigned at birth was female and that their gender identity was female. All health care providers identified as White. Health care providers included 3 registered nurses and 4 allied health professionals. All health care providers had experience delivering care to survivors of childhood cancer; 29% (2/7) of the participants had between 1 and 5 years of experience, 43% (3/7) of the participants had between 5 and 10 years of experience, 14% (1/7) of the participants had between 10 and 15 years of experience, and 14% (1/7) of the participants had >15 years of experience. Health care providers were from Alberta (2/7, 29%), British Columbia (3/7, 43%), Manitoba (1/7, 14%), and Ontario (1/7, 14%). All health care providers reported residing in urban regions. A summary of participant demographic characteristics is provided in Table 1.

**Table 1:** Demographic and clinical characteristics of survivors of childhood cancer (n=22) and demographic and professional characteristics of the health care providers of survivors of childhood cancer (n=7).

Demographic and characteristics					Value
**Survivors of childhood cancer: demographic and clinical characteristics**					
	Current age (y), mean (SD)			29.19 (4.78)	
	**Sex, n (%)**				
		Male	1 (5)		
		Female	21 (96)		
	**Gender, n (%)**				
		Male	1 (5)		
		Female	21 (96)		
	**Ethnicity^a^, n (%)**				
		Indigenous, First Nations, Inuit, or Métis	2 (9)		
		Black, African, or Caribbean	1 (5)		
		East Asian	2 (9)		
		White or European	19 (86)		
		Other	2 (9)		
	**Province of residence, n (%)**				
		Alberta	10 (46)		
		New Brunswick	1 (5)		
		Nova Scotia	4 (18)		
		Ontario	5 (23)		
		Quebec	1 (5)		
		Saskatchewan	1 (5)		
	**Geographic region, n (%)**				
		Rural	4 (18)		
		Urban	18 (82)		
	**Distance from major urban center (km), n (%)**				
		0	11 (50)		
		1-20	4 (18)		
		21-40	2 (9)		
		>40	3 (14)		
		No response	2 (9)		
	Age at diagnosis (y), mean (SD)			10.59 (5.45)	
	Time after treatment (y), mean (SD)			17.45 (6.81)	
	**Cancer diagnosis, n (%)**				
		Leukemia (eg, acute lymphoblastic leukemia and acute myeloid leukemia)	11 (50)		
		Lymphoma (eg, Hodgkin and non-Hodgkin)	6 (27)		
		Solid tumor (eg, Wilms tumor and osteosarcoma)	5 (23)		
**Health care provider: demographic and professional characteristics**					
	**Sex, n (%)**				
		Male	1 (14)		
		Female	6 (86)		
	**Gender, n (%)**				
		Male	1 (14)		
		Female	6 (86)		
	Ethnicity: White or European, n (%)			7 (100)	
	**Profession, n (%)**				
		Registered nurse	3 (43)		
		Allied health professional	4 (57)		
	Time in profession (y), mean (SD)			22.43 (8.00)	
	**Years delivering care to survivors of childhood cancer, n (%)**				
		>1 but <5	2 (29)		
		>5 but <10	3 (43)		
		>10 but <15	1 (14)		
		>15	1 (14)		
	**Province of residence, n (%)**				
		Alberta	2 (29)		
		British Columbia	3 (43)		
		Manitoba	1 (14)		
		Ontario	1 (14)		
	Geographic region: urban, n (%)			7 (100)	
	**Distance from major urban center (km), n (%)**				
		0	2 (29)		
		1-15	1 (14)		
		>15	0 (0)		
		No response	4 (57)		

^a^Participants were able to select all that apply. Total responses may exceed the total number of participants. Those that indicated “other” were invited to specify if they desired. In this sample, participants did not specify “other.”

### Priority Components to Be Included in an mHealth Intervention for Survivors of Childhood Cancer and Health Care Providers

#### Connections: Establishing Connections Is a Top Priority

##### Survivors’ Perspective

Survivors spoke of the importance of being able to connect with other survivors because they feel validated by those who understand that the cancer journey does not end when treatment ends and that, in fact, the cancer experience is lifelong (quote 1). Survivors also felt that the ability to communicate with other survivors on mHealth would allow them to establish a sense of community through a shared experience and shared understandin*g* of having been impacted by cancer (quotes 2 and 3). Survivors specified that such a connection could be a therapeutic process as well as an opportunity to provide peer support (quotes 4 and 5). Survivors also discussed the possibility of being able to connect with their health care provider through additional means outside of clinical care (quote 6). Finally, survivors emphasized that being able to establish these connections with other survivors of childhood cancer as well as health care providers was a means of self-empowerment and self-advocacy because they could take control of their own care and experiences (quotes 7-9).

##### Health Care Providers’ Perspective

Health care providers also recognized the importance of connecting survivors with one another. Health care providers shared that these social connections are likely to mitigate feelings of isolation for survivors in their cancer experience and, in turn, empower them to engage in their follow-up care (quotes 10 and 11). Health care providers also expressed a desire to connect with other health care providers across Canada for the opportunity to consult and learn from one another about pertinent matters in delivering follow-up care for survivors of childhood cancer (quote 12). In addition, health care providers shared that being able to connect with survivors (eg, navigating appointments and/or scheduling), as well as with primary care providers, may be a useful feature to incorporate in mHealth to enhance the efficiency with which they deliver follow-up care (quote 13; Textbox 1).

**Theme, subthemes, and representative quotes from survivors (n=22) and health care providers (n=7) on the development of an mHealth intervention to enhance follow-up care. This textbox covers theme (1), “connections: establishing connections is the top priority.”**

**Survivors’ perspective**

- Connecting with other survivors feels validating because not everyone understands the cancer journey and that it is lifelong.
  Quote 1: “...I think in just hearing others communicate about their experiences to speak to the volume and importance of it’s not done after, we’re not done after treatment, it’s a lifelong thing that we’ll always re-engage in at various points in time in different ways.” [S6F2]I
  Quote 2: “...the idea of a platform is really great, especially when you think about people’s readiness to engage in that kind of stuff...So having that available when people are ready to engage gives them access and gives them a sense of control about when they are actually feeling they need that support versus when you have that follow-up first talk...” [S6F2]
  Quote 3: “And you can be surrounded with people that do care about how you’re feeling and how you’re doing but no one knows what it’s like. So being able to connect to people that have gone through the same things as you, that can validate your feelings and all of that...” [S4F5]
- Connecting with other survivors can help create a sense of community.
  Quote 4: “Almost kind of like meet-up where you could get a bunch of survivors together and like, all go for coffee or something and we’ll just hang out and talk about our stories or like how we’re moving forward with our lives or what’s going on...I think it would be really cool to see something on a bigger scale that’s perhaps all the way across the country or just something along that line, as well as having resources.” [S4F3]
  Quote 5: “And also, maybe an option to talk to people who have been through the same thing, but not talk about cancer at all...Build up a bigger community around it without feeling super nervous or weirded out by it.” [S4F3]
- Connecting with health care providers may help ensure a shared understanding of what occurs at follow-up care and increase access to health care providers.
  Quote 6: “When I said, ‘I am anxious about ABC’ or like, ‘XYZ happened to me in my life,’ did they hear that or did they just say, ‘Worried about cancer but looks good. Goodbye,’ like I want to know that they had the same thing. And it would be lovely to have some kind of ability to keep on top of that or keep track of that whether that be some variation of like there’s a passport where it’s just like, ‘We had this conversation on this date’ and then I know for future that it has been brought up or it has been talked about.” [S1F4]
- Self-advocacy is integral to being a survivor of childhood cancer, and teaching people how to empower themselves through connections and knowledge is key to their health care.
  Quote 7: “They put you kind of, you’re more involved in the whole process if you have really easy access to all the information. Like [participant 3] was saying about her binder, and how she’s sort of being involved in the process with keeping track. I think that just helps you feel like you’re taking control of your own health. And it’s like, that would help engagement.” [S2F1]
  Quote 8: “I think for some of us who are a little more like question shy when it comes to like speaking to doctors, like I know, I often find myself listening and not asking. I think maybe including information on how to like advocate for yourself as a patient would be important.” [S3F3]
  Quote 9: “I’m not 100 percent sure on how this would fit in with the platform yet, but I think that something in aftercare that is kind of missing that might be beneficial in kind of helping people to be able to advocate and engage in their care a little bit more is like—I don’t know if it’s the first appointment you ever have when you go to adult care or the last one or somewhere in-between.” [S3F4]

**Health care providers’ perspective**

- Health care providers recognize that connecting survivors with other survivors is a priority. These social connections can potentially help mitigate feelings of isolation and empower them to engage in their follow-up care.
  Quote 10: “Yeah well, I agree with the idea of a platform, it helps people to move from kind of the more insular cancer experience to broadening and connecting in the community and identifying what they have in common with other people because I think that would help people and with the focus on mitigating of effects.” [HP2]
  Quote 11: “Really help them with like social opportunities because a lot of them are so isolated socially. Some of them have a really hard time making friends, because they don’t like, it’s not like a muscle memory they learned in high school. Like where they don’t—they just have a hard time knowing how to make and maintain friendships. So that I think would be a useful sort of like, a place where people like me, across the country, could refer to get people connected socially.” [HP5]
- Health care providers want to connect with other health care providers to discuss, ask questions, and share expertise.
  Quote 12: “No. I think one interesting thing too is that we, as a long-term follow-up group, have a Canadian group that we talk about. Like we need every, say, quarterly online and discuss, you know, different issues and what are you doing here? What are you doing here? So, I think having that expertise, Canadian expertise of those groups would be good. So, you know, when this is up and running it would be great if that was presented at our group. Like, you could join our email Zoom, and you know, get everyone on the same page of engagement.” [HP6]

Health care providers indicated that navigating appointments and scheduling with survivors and communication with primary care providers may be useful features.
  Quote 13: “So, I don’t know if adding the primary care information and being able for them to amend it as it changes. I don’t know if there’s a functionality where when they see an update, they could forward that to their primary care provider. I mean I’m thinking really big here and I know it’s nearly impossible to do but, you know, there must be a way to maybe get things together even if it means, you know, they have to physically show it to their primary care provider when they go for their next visit.” [HP1]

#### Education and Information: Having a Cancer History Profile Can Be Useful

##### Survivors’ Perspective

Features that provided information on and tracking survivors’ cancer history were some of the most prevalent themes discussed by survivors. Specifically, survivors emphasized the importance of having their cancer treatment history summarized in the form of a cancer profile, along with information regarding the type of follow-up care they receive, a summary of their follow-up care visits, and a summary of past and current medications and treatments (quote 14). Participants highlighted several benefits of this feature in an mHealth intervention, including being able to recall their medical history during appointments with new health care providers, establishing a shared understanding between survivors and health care providers, addressing any gaps in communication, and making their follow-up care more accessible (quotes 15 and 16).

##### Health Care Providers’ Perspective

Health care providers identified a need for a cancer profile so that information such as treatment history (eg, cumulative doses and exposures) can be summarized in 1 hub (quote 17). Furthermore, health care providers indicated that a cancer profile may serve as an effective tool to communicate with survivors about their follow-up care, including the importance of screening for late effects, provided that the cancer profile can include a summary of potential risks of late effects related to diagnosis and treatment (quote 18). Similarly, health care providers explored the possibility of linking updated survivorship guidelines to a cancer profile, which may be informative for survivors of childhood cancer (quote 19; Textbox 2).

**Theme, subthemes, and representative quotes from survivors (n=22) and health care providers (n=7) regarding the development of a mobile health (mHealth) platform to enhance follow-up care. This textbox covers theme (2), “education and information: creating a personalized cancer profile would be helpful.”**

**Survivors’ perspective**

- A treatment summary can help with communicating with health care providers at follow-up care visits and remembering treatment history details.
  Quote 14: “Yeah, I mean, I feel like, earlier like I was saying, I have that binder which I feel like is kind of like a one stop shop. But if that was in a digital form, I feel like that would be, that would be useful. Some sort of platform where, yeah it would have some sort of resource thing, but customized to what treatment you had, what drugs specifically you were on. Something that would have—even when you go to aftercare, a digital version of your test results as well. That way it’s stored there and there’s a history you can refer back to.” [S3F1]
- Being able to track and view the history of follow-up care visits would make care more convenient, transparent, and accessible.
  Quote 15: “...I guess a lot of things are getting more digital now so just being able to have access to records, maybe possible notes of previous visits you had. So, something just to refer to would be very convenient.” [S1F1]
  Quote 16: “...let’s say if you want to refer back to a visit that you did a couple of years ago, have some notes that you can kind of refer to. Other than, kind of see how you’ve improved. And I think that would just help to refer back and then see how it’s progressed, from any concerns from past follow-ups.” [S1F1]

**Health care providers’ perspective**

- A treatment summary can help health care providers consolidate information and inform treatment planning.
  Quote 17: “I think that making sure that it has like a comprehensive treatment summary, and by that, I mean...like the significant cumulative doses, that they know what complications that they have, and then the things at risk. But I think the number one thing on the very first page after their treatment summary would be the very practical things you have to do, echoes, when’s your next test due; and to also make sure that they have a primary care physician” [HP1]
- A cancer profile may help health care providers communicate the importance of understanding and screening for late effects for survivors of childhood cancer.
  Quote 18: People have to be prepared kind of for the long haul without knowing exactly what the long haul is. But for some people we have a fairly good idea that they will like pretty significant cognitive or physical disabilities depending on what they’ve gone through. So, the challenge of figuring out how to help people with that process as it evolves.” [HP2]
- Linking updated survivorship guidelines to a cancer profile may be informative for survivors of childhood cancer.
  Quote 19: “I think it could be linked to the survivorship guidelines somehow but that the less we put and the most important stuff that we put in that will actually make it useful.” [HP1]

#### Engagement: Issues Related to Accessibility and Health Equity Impact Survivors’ Engagement in Their Follow-Up Care

##### Survivors’ Perspective

Survivors discussed the challenges related to accessing their follow-up care, referring to the ease of reaching and using health care services, and how these barriers reflected issues related to health inequities that some experience. For example, survivors felt that all survivors requiring follow-up care should be able to access their follow-up care, and yet, disparities exist in this regard (quote 20). Survivors spoke about their difficulty in accessing follow-up care due to geographic barriers, especially for those that reside in more rural or remote communities (quotes 21-23). In addition, survivors shared that features such as appointment reminders, accommodation provided for transportation, and being able to ask questions to health care providers in an mHealth intervention may be ways that can help increase access to their follow-up care (quote 24).

##### Health Care Providers’ Perspective

Health care providers recognized that health inequities exist in the current service delivery of follow-up care to survivors of childhood cancer and highlighted the need to make mHealth easily usable and accessible (quote 24). Health care providers also identified practical strategies that can help survivors engage in their follow-up care, such as displaying a list of the upcoming appointments on mHealth or outlining follow-up care in a stepwise fashion so that survivors that face cognitive effects of their treatment can be accommodated and supported in their engagement with follow-up care (quotes 25 and 26; Textbox 3).

**Theme, subthemes, and representative quotes from survivors (n=22) and health care providers (n=7) on the development of an mHealth intervention to enhance follow-up care. This textbox covers theme (3), “engagement: issues related to accessibility and health equity impact survivors’ engagement in their follow-up care.”**

**Survivors’ perspective**

- Appointment reminders, flexibility, and accommodations for transportation are ways to increase access and engagement to follow-up care.
  Quote 20: “I would love to have an appointment at a certain time and for the appointment to be like within one hour of that time rather than that’s the only thing on my day because I might be pushed four or five hours. And I understand like oncology is a field that does have emergencies and things happen and yes, like those precedent absolutely. But when I’m coming there just to get a rubber stamp that said I’ve done it and they’ve already looked at my blood work, where it definitely could’ve been a phone call, but I was spending my whole day in clinic instead, those are the things that would make it easier for me to engage more with my follow-up rather than treat it like a check box.” [S3F1]
- Health inequities exist, and people from remote or rural communities have a harder time accessing their follow-up care. Telehealth mitigates some of these challenges but not all.
  Quote 21: “But aside from that a lot of the more helpful things to engage with follow-up are things that unfortunately, unless you’re building a city from scratch, are very hard to accomplish. I would like to be near my area, I would like more my follow-up is being happening, I would like for there to either be parking or public transport that is easy to access and get me there. All of those things.” [S1F4]
  Quote 22: “And if that’s something that, like I said out here, it was shocking to live the reality that health equity is a dream, not a reality in Canada as much as we want it to be. Because like people in [city], the health care here is, it’s just not accessible, it’s brutal...I mean, I think Zoom and all of the telecommunication stuff will really help now, hopefully where people are more open to doing these sorts of things virtually, rather than having to drive four hours to [city], that sounds horrible.” [S4F5]
  Quote 23: “But just, if there was something that could address that for people who maybe can’t access care where they live remotely, and they can actually be connected with practitioners or—yeah, I don’t know. I think that’s all I wanted to say about the app.” [S3F5]
- Being able to communicate with the health care team and ask questions may help increase access to follow-up care.
  Quote 24: “I also think a message feature would probably be efficient because I think of like, if I have to call the clinic sometimes that can be a little bit of a hassle with my phone tag. But if I knew that on the App, I could just send a message saying something like, ‘appointment needs to be changed,’ whatever. ‘Can you give me a call?’ Or even just being able to do it that way completely would be useful.” [S3F1]

**Health care providers’ perspective**

- Health inequities exist, and access to care can be increased by showing survivors how to navigate mHealth and making mHealth readily available.
  Quote 24: “I think that, if I had to be very sort of realistic, I think that making sure that they have things to access is the most important. I still think that that probably would be like a big barrier to building this if we had to add that functionality [to the mHealth platform].” [HP1]
- There are practical strategies that may help survivors of childhood cancer engage in their follow-up care.
  Quote 25: “But I think the number one thing on the very first page after their treatment summary would be the very practical things you have to do, echoes, when’s your next test due.” [HP1]
  Quote 26: “Yeah, just that transition to adulthood thing of helping people see what are the normal kinds of tasks that people learn to become adults; so that’s kind of more concrete for them. Perhaps break some of those tasks down into concrete steps so it’s not so overwhelming.” [HP2]

#### Resources: Providing Personalized Resources Can Help Enhance the Survivorship Experience, Psychosocial Well-Being, and Reproductive Health

##### Survivors’ Perspective

Survivors highlighted the need for resources specific to the unique needs of cancer survivorship, highlighting that personalization helps normalize the experience of survivors of childhood cancer (quotes 27 and 28). Survivors also expressed a desire for more mental health resources. They readily endorsed the impact of diagnosis and treatment on their social, emotional, and mental well-being, especially wanting to know more about the notion of posttraumatic growth and posttraumatic stress in survivorship (quotes 29-30). Survivors also highlighted major gaps in resources dedicated to reproductive health, even though there is a negative impact of cancer treatment on fertility and family planning (quote 31). Survivors also noted that educational resources to support survivors’ transition back to school are largely absent (quote 32). Finally, survivors discussed the importance of being able to access resources that are up-to-date and reflect recent advances in science and research (quote 33).

##### Health Care Providers’ Perspective

Health care providers identified, as a priority, the need for resources dedicated to survivorship experience, financial assistance, and health care transitions (quote 34). They also identified a gap in psychosocial and educational supports (quote 35). Moreover, health care providers spoke of the need for better resourcing to help survivors understand and navigate their fertility and family planning (quote 36). Finally, health care providers discussed the importance of being able to share resources that are updated and reflect advances in research and science (quote 37; Textbox 4).

**Themes, subthemes, and representative quotes from survivors (n=22) and health care providers (n=7) on the development of an mHealth intervention to enhance follow-up care. This textbox covers theme (4), “resources: providing tailored resources can help enhance the survivorship experience, psychosocial well-being, and female and reproductive health.”**

**Survivors’ perspective**

- Offering resources on the unique survivorship experience can help to normalize being a survivor of a childhood cancer.
  Quote 27: “And like it would be nice to have a platform where you can exchange stories and have some of those anxieties assuaged of like this isn’t a unique to you experience, it’s a unique to the childhood cancer experience. Like there are other people who went through the same thing.” [S1F4]
  Quote 28: “But I think that maybe people would like to access, maybe there’s testimonials from people. If it’s about people feeling like it normalizes their situation.” [S3F5]
- Mental health resources are lacking. Survivors are interested in better understanding the impact of diagnosis and treatment on their social, emotional, and mental well-being, including experiences of posttraumatic growth and posttraumatic stress.
  Quote 29: “But what I was going to say was, I would say like social, emotional, mental health resources, just that larger I think important too. Or even contacts specifically connected to the aftercare clinic with relation to those. Whether it be social work, or child and youth, depending on the age, that type of thing.” [S3F4]
  Quote 30: “So, I think education maybe around mental health, what certain terms might mean like post-traumatic growths, what is that? Or post-traumatic stress disorder, or survivor’s guilt, education content around what kind of complications could pop up for people that have had cancer treatment as a pediatric patient.” [S7F2]
- Resources on female and reproductive health of survivors of childhood cancer is inadequate. Survivors would like to be better educated and equipped with the impact of diagnosis and treatment on their female and reproductive health.
  Quote 31: “I was never told any—I had to ask if I was going to be able to have kids. It was never brought up to me. But I mean, I don’t know, I only had chemotherapy. So, I don’t know if radiation has something to do with that, but yeah, they’ve never brought it up.” [S2F5]
- Knowing what accommodations and modifications are available to survivors of childhood cancer in their learning and education would be a helpful guide.
  Quote 32: “I think maybe for deciding where this is for me, at home schooling. Because I know when I was going through treatment they had to—I skipped out like a year and a bit. So, yeah, I guess just resource things or places to refer to, to kind of continue that education if you’re not able to be in class.” [S1F1]
- Survivors would like resources that are updated and reflect advances in research and science.
  Quote 33: “...just what’s the most up to date evidence, because I think it’s constantly evolving, so I think that’s a huge piece of that information need and education need. I think it also helps you problem-solve, too, when things come up and you’re ‘is this normal?’ And then you go to research and it’s ‘yeah, 20 percent or more end up with mental healthcare needs. OK, it’s not abnormal for me to be feeling this way or for me to have this challenge.’ So, I think there’s a normalization element to having information that can support you in navigating follow-up care, for sure.” [S6F2]

**Health care providers’ perspective**

- Resources dedicated to the survivorship experience, financial assistance, and health care transitions are priorities.
  Quote 34: “So, if we can figure out parking, just sort of if you think about another practical suggestion, how do you engage, if you need financial assistance with parking or travel, links to some resources that might be helpful. Not that there’s not many, but just sort of how to eliminate barriers.” [HP4]
- Health care providers identified a major gap in psychosocial and educational supports.
  Quote 35: “Well, the big mental health ones I think is key, mental health, like the, I know [location] Health Services have their, has their cope, hard, coping in hard times kind of link or something, just those would be I think standard, would be helpful to have that. And even counselling specific, psychosocial counselling, counselors that are, identify, have experience in working in this area. Private ones would be helpful, although, yeah, and then just financial resources as well too, just links to that. I know, you could kind of there’s a whole bunch of different ones I’m sure that you could link there, but the counselling, vocational supports.” [HP4]
- Better resourcing is needed to help survivors of childhood cancer understand and navigate their fertility and family planning.
  Quote 36: “You know some common big things that, not so much for our young patients for our adolescent and young adults, I do think that sort of resources about fertility and family planning needs to be somewhere in there because I think that that’s something that some patients whether it be cultural or just personal can’t openly ask, and I think that we’ll agree that that’s probably the most complex late effect that we deal with from a physical and mental-health perspective. I think that that—I don’t know if it has to be something on its own, but I wish we could be better at telling a young person that, you know, family planning is different for everybody and that there are different ways to have families and the definition of family is changing every day. So, I don’t know if that part is just—if I won $10 million I would put the money toward fertility salvage in survivors; that’s what I would do.” [HP1]
- Health care providers would like resources that are updated and reflect advances in research and science.
  Quote 37: “Also, a place where they could see where there’s a change in practice, like how often we do echoes or, you know, new recommendations for vaccinations, you know, like the very specific stuff that they, you know—flags or alerts I think would be helpful.” [HP1]

#### Personalization: There Is a Need for Personalized Features on mHealth, Including Consideration of Survivors’ Readiness; Emotional Impact of Accessing Follow-Up Care; Privacy; and Right to Accurate, Moderated Information

##### Survivors’ Perspective

Survivors highlighted several important considerations to help personalize the mHealth intervention. For instance, survivors described the importance of providing options so that survivors can feel that they have a choice in when and how they wish to navigate mHealth in their cancer journey (quotes 38 and 39). Furthermore, survivors discussed the emotional impact of accessing their follow-up care, highlighting that the survivorship experience is diverse and that they want to be able to establish their own boundaries in accessing their follow-up care and cancer history through mHealth (quote 40). In addition, survivors discussed the importance of incorporating privacy features to protect their identity and health information, whether that is from their family members and/or other members of the mHealth community (quotes 41 and 42). Finally, survivors recognized that, while they would value being able to communicate with other survivors in a forum on mHealth, there is a need to modulate all communication to ensure that any information or resources shared are accurate and not to be mistaken with professional recommendations (quote 43).

##### Health Care Providers’ Perspective

Health care providers expressed their concern for any information that survivors receive that is not communicated directly from a health care provider (quote 44). They expressed a desire to ensure that survivors receive accurate knowledge and resources to support their follow-up care (quote 44). Health care providers also shared their concern for the limited capacity of current health care providers in being able to support the growing population of survivors (quote 45; Textbox 5).

**Themes, subthemes, and representative quotes from survivors (n=22) and health care providers (n=7) on the development of an mHealth intervention to enhance follow-up care. This textbox covers theme (5), “personalization: there is a need to account for personalized features, including survivors’ readiness; emotional impact of accessing follow-up care; privacy; and right to accurate, moderated information, in accessing mHealth.”**

**Survivors’ perspective**

- Being able to choose when to access, and have control over access, mHealth is an important feature. Not all survivors feel ready or want to engage in their mHealth at all times.
  Quote 38: “And I don’t think that it should be alert based, or maybe if there’s a way that you opt out of alerts, because I think that that would be probably a stressor, when you’re at work, and you like, ‘Ping. Have you thought about cancer today?’” [S3F5]
  Quote 39: “...the idea of a platform is really great, especially when you think about people’s readiness to engage in that kind of stuff. Oftentimes...I wasn’t ready at first to engage with other youths at that time. It took some time, and it actually was, I made my closest friendships with other survivors five years post, maybe even more than that. So, it was—I was quite defended from that, but there was a readiness there, that I was ready to do that, but it came at an appropriate time. So having that available when people are ready to engage gives them access and gives them a sense of control about when they are actually feeling they need that support versus when you have that follow-up first talk, it’s ‘hey, here are the resources available to you, have at them when you need them,’ and that can be a little intimidating.” [S6F2]
- For survivors, there is an emotional impact to accessing their follow-up care. These experiences are diverse, and survivors want to be able to establish their own boundaries in accessing their follow-up care and cancer history.
  Quote 40: “But my concern with peer support has always been that the people who tend to gravitate toward looking for support, are people who aren’t doing well. And so, I don’t want to go or be part of something that becomes a co-rumination.” [S2F5]
- Establishing privacy features is important for survivors to protect their identity and health information from their family and others.
  Quote 41: “And I think it’s not just me that you’re kind of targeting with a platform like this, it’s also especially for people who might be younger and still living at home. You’re now getting the whole family. Because, with iPads and phones and emails and all these things now, if folks are still living at home, their parents may also be seeing these notifications.” [S4F5]
  Quote 42: “I’d love to meet up with other individuals that have shared my experience one on one or even in a small group setting like this, whether it be like an optional pen pal thing through the platform where you could talk one on one. And, you know, if you wanted to talk to them through video that is your choice but make something so it’s secure so you’re not giving out personal information if you don’t want to.” [S4F5]
- It is important that any communication on mHealth is moderated and not to be mistaken with medical or professional advice.
  Quote 43: “...I do kind of feel as though there might need to be some sort of like regulation on what is said. As much as like 100 percent lived experiences and what people have gone through is so important and can be relayed, but I just would also worry that things would be maybe not communicated clearly and I would just worry that like, ‘Oh, someone said that high dose vitamin C treatments at this random clinic did something.’ And just that is the only like little thing in my ear that’s saying like just be careful is all.” [S3F5]

**Health care providers’ perspective**

- Any information shared with survivors of childhood cancer needs to be properly communicated by a health care provider.
  Quote 44: “You know I think it’s a good idea to have some sort of platform where survivors can [pause] I can’t think of the right word, that they can look at and give them information. I do sometimes think I worry if they get information without back-up to discuss it and all, like you can’t just tell somebody, ‘You’re going to be infertile’ and not go through it all, you know, discuss your options and that sort of thing. That’s what I’m thinking from that perspective. [HP3]
- There is a concern for the capacity of health care providers to be able to serve the growing population of survivors of childhood cancer.
  Quote 45: “I mean, yeah, it’s, like I said, the big piece is just increasing capacity and then, yeah, looking at, if we’re going to continue on this trend in terms of survivorship. And if we’re looking at having the seeing people indefinitely within [location], I don’t know if that’s the case, then what’s the future hold...That’s what my concern is, is that we’re really, are we really providing the best service possible if we’re, we have these volumes and we just don’t have the staff to really provide that support, that’s my main concern, I guess, where are things going and is there going to be capacity to support it.” [HP4]

### Validating Findings Facilitated Through Community Engagement

A total of 31 participants, including survivors of childhood cancer (n=10, 32%) and their caregivers (n=2, 6%), health care providers (n=6, 19%), researchers (n=11, 35%, including 2 patient partners), and collaborators (n=2, 6%) participated in the community engagement event to review and verify the qualitative results gathered.

#### Small and Large Group Discussions

**Figure 1:**
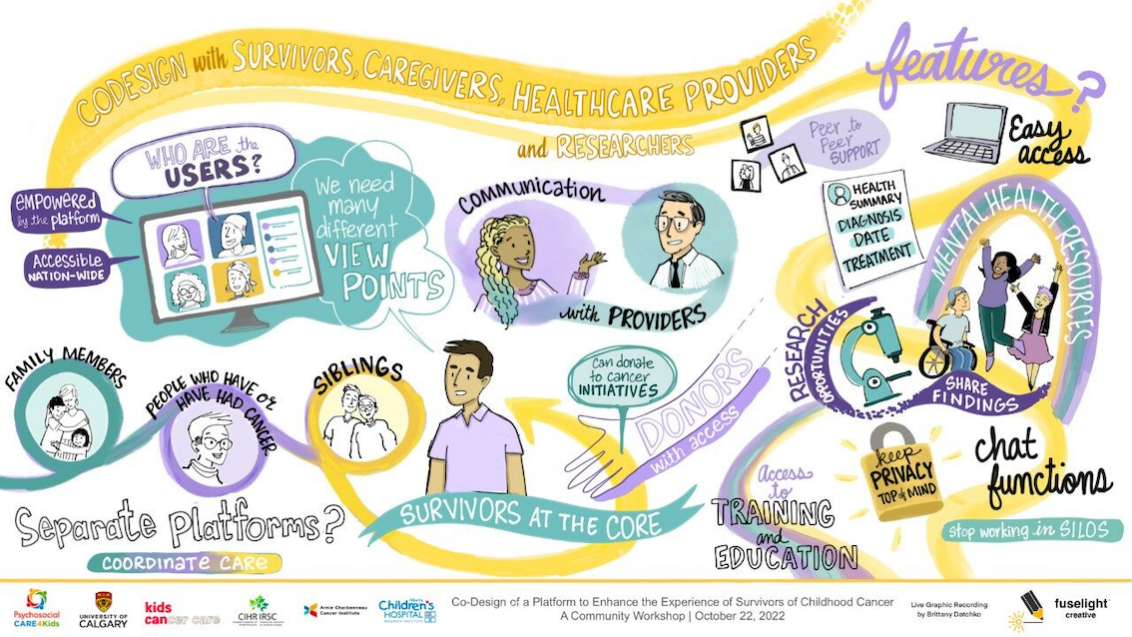
Graphic recording of the community engagement event on October 22, 2022. Illustration completed by Fuselight Creative.

Participants endorsed the accuracy of themes derived regarding the core components to be included in mHealth. Participants explored additional contexts in the development of an mHealth intervention, including considerations for family members (eg, families and siblings) and the practicalities of preserving privacy and confidentiality on a technology-based intervention. Participants also reported feeling heard through the graphic recording process. The final graphic recording is shown in Figure 1.

#### Satisfaction Survey

Some participants (8/22, 36%) completed a web-based questionnaire assessing participant engagement at the event. On average, participants *strongly agreed* that they felt that their views were heard (mean 5.00, SD 0.00) and that they were able to discuss their views freely (mean 4.71, SD 0.45). Participants also strongly agreed that they felt they had enough information to contribute to the topics discussed (mean 4.71, SD 0.35) and that a wide range of views on the topics were shared (mean 4.71, SD 0.45). Participants *mostly agreed* that they had a clear understanding of the purpose of the community workshop (mean 4.14, SD 0.35). In addition, participants were asked to indicate the extent to which they agreed that the community engagement event objectives (ie, to co-design mHealth, to engage with others, and to provide feedback on study results) were achieved, and their responses ranged from *mostly* to *strongly* agreed *(*mean scores ranging from 4.00 to 4.71, SD scores ranging from 0.45 to 0.83). Importantly, participants *strongly agreed* that they felt confident that the input provided through this event would be incorporated by the study team. A summary of participant responses is reported in Tables 2 and 3.

**Table 2:** Participant responses to community engagement event evaluation (n=8)^a^ for section 1 (overview of the workshop)^b^.

Overview of the workshop	Responses, mean (SD)
“I had a clear understanding of the purpose of this community workshop.”	4.14 (0.35)
“I had enough information to contribute to the topics discussed.”	4.86 (0.35)
“I was able to express my views freely.”	4.71 (0.45)
“I felt that my views were heard.”	5.00 (0.00)
“A wide range of views on the topics discussed were shared.”	4.71 (0.45)

^a^A total of 8 attendees, including 4 survivors of childhood cancer, 1 caregiver, 2 family members, and 1 researcher, completed the evaluation.

^b^Attendees were asked to rate the extent to which they agreed with statements in this table, using a rating scale where 1=strongly disagree and 5=strongly agree.

**Table 3:** Participant responses to community engagement event evaluation (n=8)^a^ for section 2 (workshop objectives)^b^.

Workshop objectives	Responses, mean (SD)
“To describe the preliminary results from the study on improving follow-up care for survivors of childhood cancer.”	4.14 (0.83)
“To co-design a platform for survivors of childhood cancer that is agreed upon within this community.”	4.00 (0.76)
“To engage with other core community members (health care professionals, researchers, families, and caregivers).”	4.71 (0.45)
“I am confident that the input provided through this community workshop will be used by the CARE4Kids team at the University of Calgary.”	4.71 (0.45)

^a^A total of 8 attendees, including 4 survivors of childhood cancer, 1 caregiver, 2 family members, and 1 researcher, completed the evaluation.

^b^Attendees were asked to rate the extent to which they agreed that the objectives listed in this table were achieved, using the rating scale where 1=strongly disagree and 5=strongly agree.

## Discussion

### Principal Findings

It is important that survivors of childhood cancer receive routine health care follow-up, yet many survivors do not understand the importance of their follow-up care or have limited knowledge on their need for follow-up care. Our study aimed to amplify the voices of young adult survivors of childhood cancer in identifying the priority components to be included in an mHealth intervention that can help educate and engage survivors in their long-term follow-up care.

Our approach was unique in using co-design and a qualitative research framework and adhering patient-oriented research principles to engage in mHealth intervention development [21,37]. We uniquely incorporated perspectives from survivors of childhood cancer and health care providers that deliver cancer-specific follow-up care. Patient partners collaborated with our team over the entirety of the research process, including study design, recruitment, data collection (ie, cofacilitation of focus groups and interviews), interpretation of results, and knowledge dissemination. Furthermore, a community engagement activity was cofacilitated with patient partners to enhance the validity of qualitative descriptive data gathered.

Limited mHealth interventions have been created *with* survivors of childhood cancer despite its rising potential to improve health [38]. Most efforts have focused on patients who are on active cancer treatment. For instance, a systematic review indicated a positive effect of mHealth interventions on improving the health-related quality of life of adult patients with cancer [38]. Other studies have explored delivering a survivorship care plan and an app for enhancing self-management for adolescents and young adults [39]. Few studies have effectively engaged in the co-design of mHealth, and even less work has been conducted with young adult survivors of childhood cancer in this regard, as well as with health care providers. Therefore, our work provides important and novel insights from the perspectives of individuals with lived experience of cancer and health care providers regarding their follow-up care experiences. Specifically, results shed light on the priority areas necessary to increase knowledge of and engagement in follow-up care for survivors of childhood cancer, establishing a critical foundation in mHealth intervention development.

In total, 5 major themes were conceptualized as the priority components of the mHealth intervention. Many of the themes are consistent with the existing literature on the unmet needs of survivors of childhood cancer. For example, survivors and health care providers from our study identified a lack of knowledge of diagnosis and treatment, as well as associated late effects, for survivors. These results are consistent with past work showing that most survivors of childhood cancer fail to recognize their risk for developing a serious health condition [11,40]. Furthermore, previous research indicates that survivors of childhood cancer need a better way to learn about and engage in their own health information [10,41]. Indeed, our findings indicate a need for advanced means to deliver follow-up care knowledge, such as the use of a mobile app or website, as well as the need for creative features to enhance the follow-up care experience, such as reminders for appointments and a cancer profile to consolidate health history information.

Another notable theme generated was the need for more education and support during health care transitions. This builds on an extensive body of literature on health care transitions for young adults impacted by cancer [42]. There are cancer-specific risks and health care needs of survivors of childhood cancer that are distinct from those with other chronic illnesses [43]. Therefore, our research lends further support for the importance of personalized care for young adult survivors during their health care transition from pediatric to adult health care, one that prioritizes educating survivors on their potential late effects related to their diagnosis and treatment, as well as the utility of engaging in consistent surveillance to promote longer-term optimal outcomes.

Survivors expressed some hesitation toward having regular access to their cancer history information and therefore discussed the importance of being able to have choice and control in how they navigate mHealth. These findings are consistent with existing work documenting experiences related to posttraumatic stress experienced by survivors [44] and the importance of prioritizing a trauma-informed approach in intervention development.

Several other themes were generated from this study that are novel and relevant to building an mHealth intervention for survivors of childhood cancer. Survivors and health care providers emphasized the importance of establishing connections with other survivors and health care providers. These results likely reflect a sense of disconnect that survivors experience and a desire to broaden opportunities for meaningful connections. Indeed, survivors spoke about the need to explore their identity beyond their cancer journey. Taken together, these results are consistent with past work documenting that adolescents and young adults with cancer experience substantial psychosocial challenges, including peer and family relationships and personal growth stresses [45]. Likewise, health care providers echoed the importance of cultivating connections among survivors to address feelings of isolation on their cancer journey. Importantly, health care providers also highlighted a desire to connect with other health care providers that provide follow-up care across Canada, emphasizing the importance of connecting with and consulting one another to stay informed of current concerns and practices in survivorship. Few studies have addressed the unmet needs of health care providers in delivering follow-up care to survivors of childhood cancer [23]. Our study provides unique insight of both survivors and health care providers, offering a more contextualized understanding of how to improve follow-up care from multiple perspectives.

Issues related to accessibility of follow-up care and health equity were a prominent finding from our research. These outcomes contribute to our understanding of some of the geographic barriers faced by survivors from rural or remote regions, as reflected in our study sample. Developing an mHealth intervention will aim to address these barriers by connecting survivors to their follow-up care through technology. However, barriers to health care are complex and dynamic and require consideration of factors beyond the individual, including health care providers and health care systems factors, to alleviate health disparities [46]. Therefore, further work is needed to capture the complete and intersecting effects of accessibility factors, as well as other inequities faced by survivors of childhood cancer, on their receipt of high-quality health care. These are important considerations in intervention development to ensure that the burden of change is not placed solely on survivors of childhood cancer but rather recognized as a systemic problem that requires multilevel intervention.

Our research incorporated a response validation technique, member checking, to enhance the rigor of qualitative data gathered. Previous qualitative work using member checking lacked detail and discussion in the implementation of the technique. Absence of this reporting may be confounded by epistemological and methodological challenges [36]. In this research, we conducted a comprehensive assessment and report of member checking using a multiinformant, multimodal approach to strengthen the credibility and validity of data gathered. The strength of this approach is to demonstrate the true and iterative process that we took to achieve consensus among researchers and those with lived experiences in the research process. Our goal with this undertaking is to enhance the transparency, accessibility, and replicability of best practices in qualitative health research.

### Limitations and Future Directions

We review several important limitations to be considered when interpreting the results of this study. A major strength of this research was leveraging technology for participation (ie, online focus groups and community engagement). This meant an increase in accessibility to those residing in remote or rural regions. However, reliance on technology also meant that participants from lower levels of income, or some individuals from geographically more remote or rural regions of Canada, may face greater barriers to participating because they are less likely to have access to technology. Future research incorporating community outreach, phone-based participation, or compensated travel to the local context can help to mitigate this challenge in research recruitment. Input on how we can account for potential technological limitations in reaching individuals from more remote or rural regions of Canada will be important as we build the intervention.

Research shows that a diversity of perspectives drives innovation [47]. Participants from our study offered important insights into accessibility issues that contribute to health inequities for survivors of childhood cancer. However, our sample nonetheless lacked representation and voice from individuals from diverse backgrounds (eg, diversity in ethnicity, gender identity, sex, language use, and geographic regions) and/or who are not engaged in their follow-up care. We recognize that a potential bias of our sample is that most of our sample reported attending their follow-up care. We are missing the voices of individuals who are not engaged in their care at this time, and future research leveraging purposive sampling of those not regularly attending their follow-up care would offer us important insights into the barriers they face in attending care. In addition, there is a notable disparity in gender representation of participants, with 86% (19/22) of participants identifying as female gender. Studies have found that female gender predicted attendance to follow-up care, such that female participants were more likely than male participants to attend follow-up care [48]. This bias in our sampling reinforces that our findings reflect the views of those who are attending their follow-up care. In addition, there are many factors that contribute to a lack of gender representation in cancer research, including researcher bias, gender stereotypes, and unequal social opportunities [49]. Importantly, a review of gender representation trends in psychosocial survivorship research showed that there is a trend toward a more balanced representation of men and women over a 15-year period (2007 and 1992) [49]. Taken together, our skewed sample indicates an important need to continue to engage with survivors from equity-deserving groups, particularly given the increasing diversity of the Canadian population.

Research shows that members of equity-deserving groups face significant barriers to accessing high-quality and accessible health care [50,51]. Without knowledge of the perspectives and experiences of these individuals, we are missing critical information that can help to address health disparities and, in turn, bring greater awareness to the importance of follow-up care surveillance and attendance for those from underrepresented and underserved communities. Implementation of safe, inclusive, and culturally responsive recruitment strategies is needed to increase representation in pediatric cancer research [52].

Finally, this study solicited feedback from participants and other important parties to enhance the credibility and validity of the results. We demonstrated rigor of data by using a multiinformant and multimodal approach to achieve consensus. However, the response rate for completing the satisfaction survey for our community event was 36%, so responses gathered on patient engagement may not be representative of feedback from most attendees. Another limitation of our community engagement event is that we did not determine whether attendees included those who previously participated in the focus groups and interviews. Therefore, the feedback gathered at the community event may be influenced by participants’ familiarity with the study. Future research establishing and implementing a comprehensive and systematic evaluation of patient and public engagement is necessary to strengthen the rigor of our evaluative framework and enhance patient engagement and research.

The next step of this multiphased project is to build an innovative and accessible mHealth intervention prototype based on the core components identified and grounded in an established conceptual framework for co-design of intervention development. Results from this study have provided the bedrock to progress in our development of an mHealth intervention for survivors of childhood cancer to enhance their knowledge of and engagement in their follow-up care.

### Conclusions

In this study, we identified core components to be included in an mHealth intervention to increase the knowledge of and enhance follow-up care engagement for survivors of childhood cancer. We engaged in a rigorous and iterative co-design process with survivors of childhood cancer and health care providers. We incorporated a community engagement event to validate our findings with a broader audience of community members. Findings will inform the next phase of our multiphased, co-design project, ultimately aiming to improve follow-up care and long-term outcomes for survivors of childhood cancer.

## Appendix

Multimedia Appendix 1 Focus group or interview prompts for survivors and health care providers.

Multimedia Appendix 2 Researcher’s reflexivity statements.

## Abbreviations

CIHR: Canadian Institutes of Health Research
mHealth: mobile health
REDCap: Research Electronic Data Capture
SPOR: Strategy for Patient-Oriented Research

## Data Availability

Data generated or analyzed during this study are available from the corresponding author on reasonable request.

## References

[1] (2022). New WHO report highlights scale of childhood cancer inequalities in the European region. World Health Organization.

[2] Childhood Cancer Survivor Canada homepage. Childhood Cancer Survivor Canada.

[3] Landier W, Bhatia S, Eshelman DA, Forte KJ, Sweeney T, Hester AL, Darling J, Armstrong FD, Blatt J, Constine LS, Freeman CR, Friedman DL, Green DM, Marina N, Meadows AT, Neglia JP, Oeffinger KC, Robison LL, Ruccione KS, Sklar CA, Hudson MM (2004). Development of risk-based guidelines for pediatric cancer survivors: the Children's Oncology Group Long-Term Follow-Up Guidelines from the Children's Oncology Group Late Effects Committee and Nursing Discipline. J Clin Oncol.

[4] Hudson MM, Bhatia S, Casillas J, Landier W, Section on Hematology/Oncology‚ Children’s Oncology Group‚ American Society of Pediatric Hematology/Oncology (2021). Long-term follow-up care for childhood, adolescent, and young adult cancer survivors. Pediatrics.

[5] Bhakta N, Liu Q, Ness KK, Baassiri M, Eissa H, Yeo F, Chemaitilly W, Ehrhardt MJ, Bass J, Bishop MW, Shelton K, Lu L, Huang S, Li Z, Caron E, Lanctot J, Howell C, Folse T, Joshi V, Green DM, Mulrooney DA, Armstrong GT, Krull KR, Brinkman TM, Khan RB, Srivastava DK, Hudson MM, Yasui Y, Robison LL (2017). The cumulative burden of surviving childhood cancer: an initial report from the St Jude Lifetime Cohort Study (SJLIFE). Lancet.

[6] Michel G, Mulder RL, van der Pal HJ, Skinner R, Bárdi E, Brown MC, Vetsch J, Frey E, Windsor R, Kremer LC, Levitt G (2019). Evidence-based recommendations for the organization of long-term follow-up care for childhood and adolescent cancer survivors: a report from the PanCareSurFup Guidelines Working Group. J Cancer Surviv.

[7] Reynolds K, Spavor M, Brandelli Y, Kwok C, Li Y, Disciglio M, Carlson LE, Schulte F, Anderson R, Grundy P, Giese-Davis J (2019). A comparison of two models of follow-up care for adult survivors of childhood cancer. J Cancer Surviv.

[8] American Academy of Pediatrics Section on Hematology/Oncology Children's Oncology Group (2009). Long-term follow-up care for pediatric cancer survivors. Pediatrics.

[9] Edgar AB, Borthwick S, Duffin K, Marciniak-Stepak P, Wallace WH (2012). Survivors of childhood cancer lost to follow-up can be re-engaged into active long-term follow-up by a postal health questionnaire intervention. Eur J Cancer.

[10] Oeffinger KC, Mertens AC, Hudson MM, Gurney JG, Casillas J, Chen H, Whitton J, Yeazel M, Yasui Y, Robison LL (2004). Health care of young adult survivors of childhood cancer: a report from the Childhood Cancer Survivor Study. Ann Fam Med.

[11] Kadan-Lottick NS, Robison LL, Gurney JG, Neglia JP, Yasui Y, Hayashi R, Hudson M, Greenberg M, Mertens AC (2002). Childhood cancer survivors' knowledge about their past diagnosis and treatment: childhood cancer survivor study. JAMA.

[12] Adolescents and young adults with cancer. National Institutes of Health National Cancer Institute.

[13] Docherty SL, Kayle M, Maslow GR, Santacroce SJ (2015). The adolescent and young adult with cancer: a developmental life course perspective. Semin Oncol Nurs.

[14] Szalda D, Pierce L, Hobbie W, Ginsberg JP, Brumley L, Wasik M, Li Y, Schwartz LA (2016). Engagement and experience with cancer-related follow-up care among young adult survivors of childhood cancer after transfer to adult care. J Cancer Surviv.

[15] Hou SH, Schulte FS (2022). An investigation of cultural influences in survivors of paediatric cancer: a systematic review protocol. BMJ Open.

[16] Devine KA, Viola AS, Coups EJ, Wu YP (2018). Digital health interventions for adolescent and young adult cancer survivors. JCO Clin Cancer Inform.

[17] Gonzalez BD (2018). Promise of mobile health technology to reduce disparities in patients with cancer and survivors. JCO Clin Cancer Inform.

[18] Wesley KM, Fizur PJ (2015). A review of mobile applications to help adolescent and young adult cancer patients. Adolesc Health Med Ther.

[19] Ramsey WA, Heidelberg RE, Gilbert AM, Heneghan MB, Badawy SM, Alberts NM (2020). eHealth and mHealth interventions in pediatric cancer: a systematic review of interventions across the cancer continuum. Psychooncology.

[20] McCann L, McMillan KA, Pugh G (2019). Digital interventions to support adolescents and young adults with cancer: systematic review. JMIR Cancer.

[21] Domecq JP, Prutsky G, Elraiyah T, Wang Z, Nabhan M, Shippee N, Brito JP, Boehmer K, Hasan R, Firwana B, Erwin P, Eton D, Sloan J, Montori V, Asi N, Dabrh AM, Murad MH (2014). Patient engagement in research: a systematic review. BMC Health Serv Res.

[22] Berg C, Stratton E, Esiashvili N, Mertens A, Vanderpool RC (2016). Providers' perspectives of survivorship care for young adult survivors of childhood cancer. J Cancer Educ.

[23] Howard AF, Kazanjian A, Pritchard S, Olson R, Hasan H, Newton K, Goddard K (2018). Healthcare system barriers to long-term follow-up for adult survivors of childhood cancer in British Columbia, Canada: a qualitative study. J Cancer Surviv.

[24] Slattery P, Saeri AK, Bragge P (2020). Research co-design in health: a rapid overview of reviews. Health Res Policy Syst.

[25] (2018). Strategy for patient-oriented research. Canadian Institutes of Health Research.

[26] Hou S, Henry B, Schulte F (2022). An assessment of young people living beyond cancer and factors that impact their attendance of their aftercare. Proceedings of the 54th Congress of the International Society of Pediatric Oncology.

[27] Henry B, Hou S, Drummon R, Canton K, Forbes C, Reynolds K, Schulte F (2023). Adherence to follow-up care among adolescent and young adult survivors of childhood cancer in Canada. Proceedings of the 38th Annual Canadian Association of Psychosocial Oncology Conference.

[28] Colorafi KJ, Evans B (2016). Qualitative descriptive methods in health science research. HERD.

[29] Harris PA, Taylor R, Thielke R, Payne J, Gonzalez N, Conde JG (2009). Research electronic data capture (REDCap)--a metadata-driven methodology and workflow process for providing translational research informatics support. J Biomed Inform.

[30] Harris PA, Taylor R, Minor BL, Elliott V, Fernandez M, O'Neal L, McLeod L, Delacqua G, Delacqua F, Kirby J, Duda SN (2019). The REDCap consortium: building an international community of software platform partners. J Biomed Inform.

[31] Staniszewska S, Brett J, Simera I, Seers K, Mockford C, Goodlad S, Altman DG, Moher D, Barber R, Denegri S, Entwistle A, Littlejohns P, Morris C, Suleman R, Thomas V, Tysall C (2017). GRIPP2 reporting checklists: tools to improve reporting of patient and public involvement in research. BMJ.

[32] Abelson J, Tripp L, Kandasamy S, Burrows K, PPEET Implementation Study Team (2019). Supporting the evaluation of public and patient engagement in health system organizations: results from an implementation research study. Health Expect.

[33] Braun V, Clarke V (2023). Thematic analysis. APA Handbook of Research Methods in Psychology: Research Designs: Quantitative, Qualitative, Neuropsychological, and Biological.

[34] Sandelowski M, Barroso J (2003). Classifying the findings in qualitative studies. Qual Health Res.

[35] NVivo 14. Lumivero.

[36] Birt L, Scott S, Cavers D, Campbell C, Walter F (2016). Member checking: a tool to enhance trustworthiness or merely a nod to validation?. Qual Health Res.

[37] Frisch N, Atherton P, Doyle-Waters MM, MacLeod ML, Mallidou A, Sheane V, Ward J, Woodley J (2020). Patient-oriented research competencies in health (PORCH) for researchers, patients, healthcare providers, and decision-makers: results of a scoping review. Res Involv Engagem.

[38] Buneviciene I, Mekary RA, Smith TR, Onnela JP, Bunevicius A (2021). Can mHealth interventions improve quality of life of cancer patients? A systematic review and meta-analysis. Crit Rev Oncol Hematol.

[39] King-Dowling S, Psihogios AM, Hill-Kayser C, Szalda D, O'Hagan B, Darabos K, Daniel LC, Barakat LP, Fleisher L, Maurer LA, Velázquez-Martin B, Jacobs LA, Hobbie W, Ginsberg JP, Vachani CC, Metz JM, Schwartz LA (2021). Acceptability and feasibility of survivorship care plans and an accompanying mobile health intervention for adolescent and young adult survivors of childhood cancer. Pediatr Blood Cancer.

[40] Shuldiner J, Shah N, Corrado AM, Hodgson D, Nathan PC, Ivers N (2022). Determinants of surveillance for late effects in childhood cancer survivors: a qualitative study using the theoretical domains framework. J Cancer Surviv.

[41] Lee JL, Gutierrez-Colina A, Williamson Lewis R, Wasilewski-Masker K, Meacham LR, Mertens AC, Gilleland Marchak J (2019). Knowledge of late effects risks and healthcare responsibility in adolescents and young adults treated for childhood cancer. J Pediatr Psychol.

[42] Casillas J, Kahn KL, Doose M, Landier W, Bhatia S, Hernandez J, Zeltzer LK (2010). Transitioning childhood cancer survivors to adult-centered healthcare: insights from parents, adolescent, and young adult survivors. Psychooncology.

[43] Wilkins KL, D'Agostino N, Penney AM, Barr RD, Nathan PC (2014). Supporting adolescents and young adults with cancer through transitions: position statement from the Canadian Task Force on Adolescents and Young Adults with cancer. J Pediatr Hematol Oncol.

[44] Lown EA, Phillips F, Schwartz LA, Rosenberg AR, Jones B (2015). Psychosocial follow-up in survivorship as a standard of care in pediatric oncology. Pediatr Blood Cancer.

[45] Zebrack B, Isaacson S (2012). Psychosocial care of adolescent and young adult patients with cancer and survivors. J Clin Oncol.

[46] Kilbourne AM, Switzer G, Hyman K, Crowley-Matoka M, Fine MJ (2006). Advancing health disparities research within the health care system: a conceptual framework. Am J Public Health.

[47] Díaz-García C, González-Moreno A, Jose Sáez-Martínez F (2014). Gender diversity within R & D teams: its impact on radicalness of innovation. Innovation.

[48] Klosky JL, Cash DK, Buscemi J, Lensing S, Garces-Webb DM, Zhao W, Wiard S, Hudson MM (2008). Factors influencing long-term follow-up clinic attendance among survivors of childhood cancer. J Cancer Surviv.

[49] Hoyt MA, Rubin LR (2012). Gender representation of cancer patients in medical treatment and psychosocial survivorship research: changes over three decades. Cancer.

[50] Reeves TJ, Mathis TJ, Bauer HE, Hudson MM, Robison LL, Wang Z, Baker JN, Huang IC (2021). Racial and ethnic disparities in health outcomes among long-term survivors of childhood cancer: a scoping review. Front Public Health.

[51] Bhatia S (2011). Disparities in cancer outcomes: lessons learned from children with cancer. Pediatr Blood Cancer.

[52] Duong J, Forbes C, Henry B, Bilash T, Rahamatullah I, Hou S, Garland S, Bender J, Schulte F (2023). Exploring lived experiences and inclusive engagement practices for under-represented adolescents and young adults in psychosocial oncology research: a qualitative study. Proceedings of the 38th Annual Canadian Association of Psychosocial Oncology.

