# Supplementary material for: Co-Designing Priority Components of an mHealth Intervention to Enhance Follow-Up Care in Young Adult Survivors of Childhood Cancer and Health Care Providers: Qualitative Descriptive Study
Source: JMIR Cancer. 2025 Apr 25;11:e57834. doi: 10.2196/57834 (PMC12064980; doi:10.2196/57834)
Supplement: Multimedia Appendix 1 [file cancer_v11i1e57834_app1.docx]

Supplementary File 1: Focus group/interview prompts for survivors and healthcare providers.

| **mHealth Patient Platform** | | |
| --- | --- | --- |
| Section | Question(s) for Survivors | Question(s) for Healthcare Providers |
| Content | What are your thoughts on creating a platform for survivors? | |
|  | Do you agree or disagree that creating a platform for survivors to use and interact with would help to increase understanding of / engagement in follow-up care? | |
|  | If we were to build a platform, what types of features would you want to see? | |
| Education | What type of information would you want included? | |
| Communication | Would you want an opportunity to interact with other survivors and/or healthcare providers? | Would you want an opportunity to interact with other healthcare providers and/or patients? |
| Engagement | What are some features that could help you engage in your follow-up care? | What are some features that could help you increase your engagement, and your patients’ engagement, in follow-up care? |
| Conclusion | Is there anything missing from our discussion that you want to share? | |
