# Supplementary material for: Co-Designing Priority Components of an mHealth Intervention to Enhance Follow-Up Care in Young Adult Survivors of Childhood Cancer and Health Care Providers: Qualitative Descriptive Study
Source: JMIR Cancer. 2025 Apr 25;11:e57834. doi: 10.2196/57834 (PMC12064980; doi:10.2196/57834)
Supplement: Multimedia Appendix 2 [file cancer_v11i1e57834_app2.docx]

Supplementary File 2: Researcher’s Reflexivity StatementsTop of FormBottom of Form

We engaged in reflexivity throughout data analysis and interpretation of results to foster self-awareness, rigorous, and transparency of our research process. We actively examined the influences of our positionality, cultural worldviews, and life experiences, on the analysis and interpretations of data collected. The analysis team was comprised of five members: SH, RD, BH, KC, HW, overseen by FS. SH is a registered psychologist and postdoctoral fellow that specializes in clinical child and adolescent psychology and pediatric health. SH’s research and clinical interests are on advancing diversity and health equity in children’s health and well-being and those of their families. SH’s work is largely influenced by her own immigration experience and exposure to cultural diversity over her life. This study was led by SH. RD is a master’s student with training in health policy, and psychosocial functioning among adolescent and young adult survivors of childhood cancer. She is also a survivor of childhood cancer and believes that leveraging community-based research methods is the best way to inform our healthcare system of the needs of individuals. BH has a BSc in Psychology and is a research coordinator. Her interests lie in improving psychosocial outcomes for those living with or beyond cancer through patient-oriented research. HW is a medical student who holds an undergraduate degree in kinesiology with additional training in psychology, and neuroscience. Her research interests focus on improving the quality of life of individuals with chronic diseases, drawing inspiration from her work with children with cancer. KM is a subspecialty resident in pediatric hematology/oncology, providing patient care for children and youth who are living with or survivors of cancer. He has an interest in optimizing patient experience through qualitative methods and narrative medicine. FS is an associate professor and a leader in psychosocial oncology. FS is the principal investigator of this study and oversaw the study process.
